# Telemedicine in the Care of Older Adults with Dementia: Caregivers’ Perceptions and Experiences

**DOI:** 10.3390/geriatrics10060169

**Published:** 2025-12-17

**Authors:** Roni Chaim Mukamal, Viviane Gontijo Augusto, Laiane Moraes Dias, Thiago Dias Sarti, Guilhermina Rego

**Affiliations:** 1 Faculty of Medicine, University of Porto, 4200-319 Porto, Portugal; guilherminarego@med.up.pt; 2 Faculty of Medicine, Federal University of Espirito Santo, Vitoria 29043-900, Espírito Santo, Brazil; tdsarti@gmail.com; 3 Geriatrics and Gerontology Service, Cassiano Antônio de Moraes University Hospital, Federal University of Espirito Santo, Vitoria 29043-260, Espírito Santo, Brazil; 4 Department of Rehabilitation and Health Sciences, University of the State of Minas Gerais (UEMG), Divinópolis 35500-000, Minas Gerais, Brazil; vivianeaugusto2013@gmail.com; 5 Joao de Barros Barreto University Hospital, Federal University of Para, Belem 66073-000, Para, Brazil; laianemdias@gmail.com

**Keywords:** telemedicine, dementia, caregivers, older adults, remote care, digital inclusion

## Abstract

**Background:** Population aging has led to a rise in dementia prevalence, increasing the demand for innovative care models. Telemedicine offers an opportunity to improve access, continuity, and caregiver support for older adults with cognitive impairment. **Methods:** This qualitative descriptive study was conducted at the Geriatrics and Gerontology Service of Cassiano Antônio de Moraes University Hospital (HUCAM-UFES), Brazil. Semi-structured interviews were carried out with 11 caregivers of older adults living with dementia who participated in telemedicine consultations. Data was analyzed thematically using a reflexive thematic analysis approach. **Results:** Caregivers considered telemedicine useful, accessible, and safe, facilitating the continuity of care and strengthening the caregiver–professional relationship. The main limitations were the absence of physical examination and occasional technical difficulties. Most caregivers favored a hybrid care model, combining remote and in-person visits. **Conclusions:** Telemedicine proved to be a feasible and well-accepted strategy for the care of older adults with dementia, improving caregiver support and communication with healthcare teams. Public policies should foster digital inclusion and training for both caregivers and professionals, consolidating hybrid, person-centered models of care.

## 1. Introduction

The global aging of the population, particularly in Latin America, has led to an unprecedented increase in age-related diseases such as dementia, elevating their significance as a public health issue [1]. Dementia is an umbrella term for a group of chronic disorders characterized by cognitive and functional impairment, encompassing a variety of specific clinical conditions, including Alzheimer’s disease, vascular dementia, Lewy body dementia, and frontotemporal dementia, among others [2].

The number of people living with dementia worldwide is projected to rise dramatically in the coming decades. According to the Global Dementia Observatory and the World Health Organization, dementia ranks as one of the leading causes of death globally and is a major cause of disability and dependence among older adults [2].

Individuals with dementia experience frequent emergency department visits, multiple care transitions, inappropriate medication use, and end-of-life care that is often not aligned with their personal values [3,4]. These factors contribute to significant suffering and financial burden for families, as well as increased costs for the healthcare system. Given the prolonged and progressive course of the disease, continuous monitoring by a specialized team is essential to support both patients and their families throughout the various stages of illness [5].

Moreover, caregivers play a central role in meeting the complex care needs of individuals with dementia, which substantially increases the responsibilities placed upon them [4,6]. The clinical presentation and behavioral disturbances associated with dementia pose multiple challenges for caregivers, often leading to frustration, psychological distress, depressive symptoms, diminished quality of life, and a negative impact on both their physical and mental health. Caregivers often experience a high burden of responsibility, which reduces their capacity for self-care [7].

Ensuring equitable access to healthcare for this population remains a major challenge in the current era. This underscores the urgent need for public policies that foster appropriate approaches and strengthen preventive strategies, integrating technology as an essential component of healthcare systems to ensure accessibility and continuity of care [8].

Telemedicine, defined as the use of internet-connected devices to facilitate communication between healthcare professionals and patients, has proven to be a valuable support tool for older adults and their caregivers, particularly those with complex needs. By facilitating home monitoring, telemedicine expands opportunities for accessing medical care. Digital health interventions offer several advantages, such as promoting autonomy and self-management, and enabling patients to maintain greater independence [9,10].

Caregivers of people living with dementia frequently report unmet needs related to guidance, emotional support, and timely access to healthcare professionals, especially as the disease progresses. Telemedicine has emerged as a promising strategy to address these demands by facilitating rapid communication with clinicians, reducing the logistical burden of transportation, and enabling closer monitoring within the home environment. Virtual care models may enhance caregiver satisfaction, support decision-making, and reduce stress associated with managing chronic and neurodegenerative conditions, while maintaining continuity of care and strengthening the caregiver–professional relationship [6,11].

The successful implementation of telemedicine depends on usability, affordability, and rigorous guarantees of data privacy and security, from the perspective of health professionals, patients, and their caregivers [10].

Although telemedicine has advanced as a healthcare strategy in recent years, few studies have examined the experiences of caregivers of older adults with dementia in its use, particularly in the Latin American context. This knowledge gap is particularly relevant given the challenges faced by this population, such as cognitive impairment, functional dependency, multimorbidity, and frailty, which add further complexity to both care provision and the use of digital technologies. Understanding caregivers’ perceptions and experiences is crucial for guiding more inclusive care practices and for developing hybrid care models that effectively integrate technology with the principles of humanized care [4].

Therefore, the present study aimed to investigate the perceptions, experiences, and challenges faced by caregivers of older adults with dementia regarding the use of telemedicine in home care. We examined communication and relationships with health professionals, perceived effectiveness, technological usability, data security and confidentiality, and willingness to continue with this care model during and after the COVID-19 pandemic, focusing on a subgroup of caregivers of older adults with a clinical diagnosis of dementia.

## 2. Materials and Methods

### 2.1. Study Design

The study sample comprised caregivers of individuals with dementia who participated in a larger qualitative and descriptive field investigation entitled “Caregivers’ Perception of the Feasibility of Using Telemedicine in the Care of Older Adults with Complex Health Needs.” Participants were recruited from the Geriatrics and Gerontology Service of the University Hospital Cassiano Antônio de Moraes, Federal University of Espírito Santo (HUCAM-UFES), Brazil. The service provides care for patients referred to from other healthcare facilities or older adults with complex needs discharged from the hospital. All patients undergo an initial assessment, including the Edmonton Frail Scale, and those identified as vulnerable or frail are followed by the geriatric team, which performs comprehensive geriatric assessment to ensure continuity of care [12].

### 2.2. Sampling and Participants

Participants were selected by convenience sampling. Inclusion criteria included: (1) caregivers of older adults with dementia who were being followed at the geriatric’s outpatient clinic; (2) caregivers who had participated in at least one teleconsultation in the three months preceding data collection; and (3) those who provided written informed consent. Of the 16 caregivers interviewed in the primary study, 11 were caring for individuals with a clinical diagnosis of dementia according to DSM-V criteria and were therefore included in this analysis. Sample size was determined by the concept of data saturation, with interviews ending when no new relevant information emerged.

### 2.3. Data Collection

Data on care recipients were extracted from electronic medical records, including information from the patients’ Comprehensive Geriatric Assessment (CGA), which provided demographic, clinical, and functional data such as dementia severity, functional status, frailty, multimorbidity, polypharmacy, and geriatric syndromes.

Semi-structured interviews were conducted via videoconference using the Microsoft Teams platform, which was also employed for teleconsultations at the institution. The interviews were conducted by undergraduate medical students, previously trained and affiliated with the university’s Academic League of Geriatrics and Gerontology, under the supervision of the study’s principal researcher (RCM). All interviews were audio-recorded, transcribed, and securely stored in the institutional cloud of the principal investigator.

The interview guide explored five main dimensions, defined in the initial study called: The role of telemedicine in enhancing palliative care for older adults: opportunities and challenges [10]. In this review, key thematic areas relevant to assessing the experiences of older adults with telemedicine were identified. The guide questions and contents were reviewed for pertinence and clarity by a committee of five expert geriatricians experienced in telemedicine, who confirmed that the questions were clear to the target population and relevant to the study objectives.

### 2.4. Dimensions Addressed

- Relationship with health professionals: Quality of communication and sense of connection established during teleconsultations.
- Perceived effectiveness of teleconsultations: Caregivers’ assessment of telemedicine’s ability to address the clinical needs of individuals with dementia.
- Technological usability: Aspects related to accessibility, ease of use, and challenges encountered when using digital platforms.
- Confidentiality and data privacy: Concerns about information security during virtual healthcare delivery.
- Attitudes toward continued telemedicine use: Willingness and attitudes regarding the future use of telemedicine as part of ongoing care.

### 2.5. Ethical Considerations

The study adhered to standards of rigor and reflexivity typical of qualitative health research, ensuring trustworthiness and representativeness of participants’ perceptions. All participants received detailed information about the research protocol and signed the informed consent form prior to the interview. The study protocol was approved by the Research Ethics Committee of the Federal University of Espírito Santo (CAAE 5.946.428) and conducted in accordance with the Declaration of Helsinki. To ensure confidentiality, participants were identified only as “Caregiver” followed by a sequential number (e.g., Caregiver 01), with no personal information disclosed in transcripts or publications.

### 2.6. Data Analysis

Transcripts were subjected to thematic analysis, guided by the framework developed by Braun and Clarke [13]. The analytical process included data familiarization, initial coding, and iterative identification and refinement of themes by a pair of independent analysts from the research team, ensuring consistency and reliability [13].

Codes were developed inductively from the interview transcripts and subsequently according to the predefined thematic domains of the interview guide. Coding occurred in parallel with data collection, and data saturation was reached after the ninth interview, when no new codes or relevant concepts emerged. The complete coding framework is presented in Supplementary Material S1. After the codes were into final thematic axes, facilitating a comprehensive analysis that captured both positive perceptions and limitations. The analysis was conducted manually based on principles of immersion and qualitative rigor, and was designed to highlight the perceptions, challenges, and adaptive strategies associated with the use of telemedicine in the care of individuals with dementia [13].

## 3. Results

### 3.1. Sociodemographic and Clinical Characteristics of Older Adults

The sample consisted of 11 older adults diagnosed with dementia, accompanied by their caregivers. There was a balanced distribution between sexes, with a slight predominance of females (55%) over males (45%). Regarding education, elementary school was most common (64%), followed by illiteracy (18%), high school (9%), and higher education (9%), reflecting the typical educational profile of the Brazilian older adult population.

Most caregivers were informal (91%), indicating that care was primarily provided by family members or close individuals without specific technical training. Among them, 91% were women, only one caregiver was male, and all informal caregivers were either adult children or partners of the older adults with dementia. All older adults exhibited multimorbidity (100%), defined as the presence of two or more chronic conditions, which underscores the clinical complexity of this population [14]. Severity of dementia was classified according to DSM-5 and the Clinical Dementia Rating (CDR) scale: 27% mild, 45% moderate, and 27% severe [15,16]. Functional dependence was universal (100%), assessed by the Katz and Lawton indices, showing significant limitations in basic and instrumental activities of daily living [17].

Polypharmacy, the use of five or more medications, was present in 91% of cases, reflecting high therapeutic complexity and risk of drug interactions [18]. Geriatric vulnerabilities were frequent: 18% had visual impairment, 36% locomotor difficulty, 45% urinary incontinence, and 36% postural instability (history of falls in the last six months) [19]. Frailty, as assessed by the FRAIL instrument, was present in 64% of the older adults [20].

Table 1 summarizes the sociodemographic and clinical characteristics.

### 3.2. Thematic Findings Across the Five Dimensions

The analysis identified specific subthemes within each of the five predefined dimensions. Table 2 summarizes the positive aspects and limitations associated with each domain.

**Dimension 1: Relationship with Health Professionals**

Caregivers described teleconsultations as characterized by clear communication, attentiveness, and a sense of trust in the healthcare team. While interaction was generally perceived as supportive, some participants noted the absence of physical presence as a limitation. These perceptions encompassed both welcoming communication and occasional feelings of distance compared to in-person encounters.

**Dimension 2: Perceived Effectiveness of Teleconsultations**

Telemedicine was considered effective for maintaining follow-up, receiving guidance, and addressing simple clinical issues. Caregivers reported that remote encounters enabled continuity of care and facilitated management of stable conditions. Limitations primarily involved situations requiring physical examination, prompting many participants to express preference for a hybrid approach.

**Dimension 3: Technological Usability**

Most caregivers reported that telemedicine was easy to access when supported by family members. Usability was influenced by the availability of devices, internet stability, and the presence of younger relatives who assisted with technology. Difficulties occurred mainly due to connection issues or limited digital skills, reflecting variability in digital literacy.

**Dimension 4: Confidentiality and Data Privacy**

Caregivers generally trusted the institution and felt comfortable sharing information during teleconsultations. Even so, some expressed concerns about potential online scams or uncertainty regarding digital security. These perceptions reflected a balance between institutional trust and broader apprehension about online environments.

**Dimension 5: Attitudes Toward Telemedicine Use**

Overall attitudes toward telemedicine were positive. Caregivers appreciated its practicality and convenience, particularly for older adults with mobility limitations. Most participants stated that they would continue using telemedicine, though they favored alternating it with in-person visits for more complex clinical assessments.

## 4. Discussion

The sample in this study consisted of clinically complex older adults with dementia, characterized by high levels of dependence, multimorbidity, frailty, and polypharmacy, as described in Section 3. This profile reflects a highly vulnerable population with multiple clinical conditions, which increases both the complexity of care and the burden placed on informal caregivers. Overall, caregivers’ perceptions of telemedicine were largely favorable, especially in the pandemic context of pandemic-related mobility restrictions. Telemedicine was viewed as an effective strategy to maintain ongoing care, preserve patient safety, and optimize healthcare resources. While recognizing modality limitations, caregivers validated telemedicine as useful, viable, and, in some situations, preferable to in-person care [10].

### 4.1. Relationship with Professionals

Most caregivers reported positive relationships with healthcare professionals during teleconsultations. They valued empathy, active listening, and accessibility demonstrated by the physician, describing the interaction as welcoming even in the virtual setting. Although the lack of physical contact was recognized as a limitation, the sense of connection was maintained.

“Even being online, it’s very close attention.”

(Interview 10)

Most caregivers emphasized the importance of maintaining a close bond with the healthcare team during teleconsultations, highlighting the professionals’ empathy, active listening, and availability, elements that help mitigate the absence of social and in-person interactions. People living with dementia often present complex medical and psychosocial needs, which can be effectively addressed through virtual care models that integrate these specific demands. A U.S. cohort study involving 18,037 patients with dementia found that older individuals and those residing farther from medical clinics were more likely to use telemedicine. Virtual consultations with older adults also facilitate discussions about personal and clinical history, mood, medication management, treatment goals, and caregiver participation, while allowing for the sharing of visual information, such as images of skin conditions or wounds [4,21].

Maintaining the bond, empathy, and attentive communication were repeatedly highlighted as ways to compensate for the absence of social presence. Nevertheless, some felt that interaction in person is irreplaceable for establishing trust and security:

“Online is good, but in person we feel more at ease.”

(Interview 1)

Kruse et al. [22] highlights the importance of in-person interaction in complex care contexts, emphasizing its irreplaceable role in establishing trust. In this study, all patients attended an in-person consultation at the geriatric service, suggesting a potential hybrid care model for this patient population, in which the initial consultation is conducted face-to-face, while subsequent follow-ups alternate between virtual and in-person visits [22].

Even in the absence of physical contact, welcoming communication was perceived as effective in transmitting trust and support. It is fundamental that health professionals provide support to the caregiver for the execution of daily activities. These findings are consistent with studies that suggest that the quality of communication and the professional’s attitude can partially compensate for the absence of in-person interaction [23]. Social presence, a concept referring to the perception of closeness, warmth, and interpersonal connection that patients and health professionals feel during a virtual interaction, was also observed in this study [24].

Another relevant aspect in this context of complex care is the physical, psychological, and biological vulnerability that many caregivers exhibit. The continuous provision of care requires dedication and predisposes the caregiver to illness. For example, self-reported depression among caregivers of people with dementia can reach 83% [25]. In-person interaction plays an important role in dementia care, suggesting that a hybrid model, with initial in-person consultation followed by telemedicine for follow-up, is optimal for this population [10,24].

### 4.2. Effectiveness

Caregivers considered telemedicine effective in maintaining continuity of care, managing chronic diseases, adjusting medication, and following protocols, especially during the pandemic’s mobility restrictions. Simple clinical demands were reportedly addressed remotely.

“Everything was resolved by the teleconsultation.”

(Interview 10)

Beyond continuity and clinical management, there are some additional mechanisms through which telemedicine improves caregiving effectiveness. Remote psychological support such as cognitive-behavioral strategies, stress-management tools, and counseling have been associated with reductions in caregiver depression, stress, and burden. Telehealth platforms also facilitate virtual support groups and peer-to-peer interaction, mitigating isolation and promoting shared coping strategies. Moreover, remote monitoring allows clinicians to assess cognitive, behavioral, and emotional changes within the home environment with good feasibility and reliability. Together, these benefits enhance caregiver well-being and strengthen their ability to manage complex care needs [26,27].

This perception is supported by reviews that document comparable clinical results between in-person and remote consultations in long-term care [10]. However, some limitations were pointed out, especially in situations that require physical examination, functional assessment, or more complex interventions. In these cases, interviewees emphasized the importance of alternating with in-person care:

“I think in-person is still not surpassable.”

(Interview 9)

Thus, the literature’s recommendation that a hybrid model is most appropriate for geriatrics and palliative care is reinforced. Telemedicine was perceived as effective in maintaining continuity of care, particularly in the follow-up of chronic conditions, therapeutic adjustments, and the reinforcement of previously established care plans. This perception aligns with evidence from systematic reviews demonstrating comparable clinical outcomes between in-person and remote consultations in long-term care settings. Nevertheless, the lack of physical examination was identified as a limitation, especially in situations requiring functional assessment or detailed physical evaluation, further supporting the recommendation that hybrid care models are preferable in geriatric and palliative contexts [10,28].

### 4.3. Usability

The usability of telemedicine was generally considered satisfactory by the participants. Most reported no need for significant adaptations in the home environment and described ease of access, especially when supported by younger family members. The presence of a technological support network (children, grandchildren, or caregivers) was decisive for the viability of consultations, as reported in previous studies [29].

“My daughter and my niece helped me.”

(Interview 3)

Although caregivers generally adapted well to telemedicine, some interviewees reported occasional difficulties related to connection instability and limitations of the platforms used, many informal caregivers are themselves older adults and may have limited digital literacy. These challenges underscore the need for more intuitive and accessible technologies, particularly for older adults and caregivers with lower levels of digital literacy, as well as the importance of offering digital training to support telemedicine use.

The experience was often facilitated by the assistance of family members, especially children and grandchildren, demonstrating that the presence of a digital support network plays a decisive role in promoting adherence. Simplifying digital platforms and providing targeted training for users with limited technological skills therefore emerge as key strategies to enhance the accessibility and effectiveness of telemedicine.

### 4.4. Confidentiality

The perception of the security of shared information was positive in most interviews.

Caregivers demonstrated trust in the professionals and institutions involved. Reports on insecurity were specific and generally associated with previous experiences with fraud or limited knowledge about digital security:

“The internet is never secure, but we trust because we don’t have many options.”

(Interview 9)

This finding aligns with studies that suggest that the perception of security is more influenced by the institution’s reputation than by technical knowledge about data protection [9].

“We see that it’s from the hospital itself, so we trust.”

(Interview 1)

Although secrecy was not a significant obstacle to adherence, digital education and transparency regarding data protection are relevant elements to strengthen confidence in this model of care.

### 4.5. Attitude

Regarding the attitude towards telemedicine, positive acceptance was observed by most caregivers. Practicality, comfort, and security were pointed out as facilitators of adherence. Adaptation has been facilitated by the widespread availability of mobile devices and a strong interest among caregivers in receiving dementia-related information and participating in care decisions [30]. Many caregivers stated that they would continue to use the remote modality whenever necessary, especially given the difficulties in locomotion of older adults with dementia:

“If necessary, yes. It avoids going out with my 86-year-old mother.”

(Interview 1)

However, the majority advocated the use of telemedicine as complementary to in-person care, especially for more complex clinical cases or for functional assessment of the patient.

“Online consultation is good, but the ideal is to alternate with in-person.”

(Interview 11)

This perception is also described in international studies on the care of older adults with neurodegenerative conditions. The preference for the hybrid model reflects the need to balance convenience and quality in clinical assessment [4].

### 4.6. Limitations

This study presents several limitations. Data were collected during the COVID-19 pandemic, a period in which restricted mobility may have increased caregivers’ acceptance of telemedicine. The absence of physical examination remained an inherent constraint of the modality, particularly for functional or complex geriatric assessments. Usability challenges were also reported, including reliance on younger relatives for technological support, occasional internet instability, and limited digital literacy among some caregivers. Although concerns about confidentiality were minor, they were influenced by prior negative online experiences and limited understanding of digital security. The study was conducted in a single specialized geriatric outpatient clinic, with a relatively small sample, and demographic information was limited to the data available in caregivers’ records, which may reduce generalizability.

## 5. Conclusions

The findings of this study indicate that telemedicine is a feasible and well-accepted alternative for caregivers of older adults with dementia, particularly for the ongoing management of chronic conditions and for providing support during home care. Telemedicine was perceived as a safe and effective modality for ensuring continuity of care, offering greater convenience and reducing the stress associated with in-person consultations. Despite limitations related to the absence of physical examination and occasional technological challenges, caregivers expressed a high level of satisfaction and confidence in remote care, recognizing its value for clinical management and its role in strengthening the relationship with the healthcare team.

### 5.1. Practical Implications

Implementing telemedicine in the care of individuals with dementia has demonstrated capacity for optimizing caregivers’ time, broadening access to specialized services, and enhancing communication among the healthcare team, patient, and family. These results reinforce the potential of telemedicine as a continuous support tool, capable of fostering greater safety, autonomy, and quality of life for both caregivers and patients. A hybrid model, alternating between virtual and in-person visits, emerges as the most suitable format to meet the clinical and emotional needs of this population, balancing the advantages of technology with the essentials of humanized care.

### 5.2. Future Recommendations

It is recommended that public policies be developed to promote digital inclusion for older adults and their caregivers, investing in technological training, improved connectivity infrastructure, and the establishment of specific clinical protocols for the use of telemedicine in dementia care. Further research should examine the long-term impacts of this care model on caregiver burden, patient clinical outcomes, and the integration of telemedicine within primary and specialized care systems. Consolidating these approaches may contribute to more equitable, sustainable, and person-centered care for older adults and their families.

## Figures and Tables

**Table 1:** Sociodemographic and clinical characteristics of older adults with dementia (*n* = 11).

Variable	Category	No. of Participants (*n* = 11)	%
Gender	Female	6	55
	Male	5	45
Education level	Illiterate	2	18
	Elementary school	7	64
	High school	1	9
	Higher education	1	9
Formal caregiver	Yes	1	9
	No	10	91
Multimorbidity	Yes	11	100
	No	0	0
Dementia severity	Mild	3	27
	Moderate	5	45
	Severe	3	27
Polypharmacy	Yes	10	91
	No	1	9
Visual impairment	Yes	2	18
	No	9	82
Locomotor difficulty	Yes	4	36
	No	7	64
Urinary incontinence	Yes	5	45
	No	6	55
Functional dependency	Yes	11	100
	No	0	0
Postural instability	Yes	4	36
	No	7	64
Frailty	Yes	7	64
	No	4	36

Note: Values are expressed as absolute numbers (*n*) and percentages (%).

**Table 2:** Positive aspects and limitations observed in the use of telemedicine by caregivers of older adults with dementia.

Thematic Axis	Positive Aspects	Limitations/Observations
Relationship with the Professional	Welcoming and attentive communication; trust; empathy even at a distance	Lack of physical contact; sometimes distant interaction
Effectiveness	Continuity of care; satisfaction with medical advice; resolution of simple issues	Difficulty in physical assessments; preference for hybrid model
Usability	Easy access with family support; adequate home environment; digital adaptation	Dependency on third parties for technology use; occasional problems with connection or application
Confidentiality and Privacy	Trust in the team and institution; no data exposure incidents	Occasional fear of virtual scams; uncertainty about digital environment security
Attitude	General acceptance of telemedicine; willingness to continue using; adherence to medical advice	Preference for alternating with in-person care; doubts about exclusive long-term use

## Data Availability

Data supporting this study is available on reasonable request from the corresponding author.

## Supplementary Materials

The following supporting information can be downloaded at: https://www.mdpi.com/article/10.3390/geriatrics10060169/s1, Supplementary Material S1: Coding Structure and Thematic Framework.
