# Supplementary material for: Telemedicine in the Care of Older Adults with Dementia: Caregivers’ Perceptions and Experiences
Source: Geriatrics (Basel). 2025 Dec 17;10(6):169. doi: 10.3390/geriatrics10060169 (PMC12733021; doi:10.3390/geriatrics10060169)
Supplement: Supplementary file 1 [file geriatrics-10-00169-s001.zip › geriatrics-4010503-supplementary.pdf]

Supplementary Material S1.

Coding Structure and Thematic Framework

Theme	Subthemes / Codes
1. Relationship with Health Professionals	• Humanized and empathic care<br>• Clear communication<br>• Previous relationship improves acceptance<br>• Teleconsultation similar to in-person<br>• Impersonal or cold interaction<br>• Lack of human presence
2. Perceived Effectiveness of Teleconsultations	• Continuity of care<br>• Clinical resolutiveness<br>• Reduced need for travel<br>• Suitable for reduced mobility<br>• Adherence to recommendations<br>• Increased sense of security<br>• Preference for hybrid model<br>• Absence of physical examination
3. Technological Usability	• Easy when functioning well<br>• Technological barriers<br>• Internet instability<br>• Dependence on family support<br>• Digital learning and adaptation<br>• Convenience and practicality<br>• Reduced risk of COVID-19 exposure
4. Confidentiality and Data Privacy	• Trust in healthcare team<br>• Institutional security perception<br>• Fear of scams<br>• Uncertainty about privacy
5. Attitudes Toward Continued Telemedicine Use	• Willingness to continue<br>• Telemedicine facilitates caregiving<br>• Preference for hybrid model<br>• Convenience and comfort<br>• Perceived usefulness
